# Big data, artificial intelligence, and structured reporting

**DOI:** 10.1186/s41747-018-0071-4

**Published:** 2018-12-05

**Authors:** Daniel Pinto dos Santos, Bettina Baeßler

**Affiliations:** Department of Radiology, University Hospital of Cologne, Kerpener Str. 62, 50937 Cologne, Germany

**Keywords:** Artificial intelligence, Information technology, Machine learning, Radiology

## Abstract

The past few years have seen a considerable rise in interest towards artificial intelligence and machine learning applications in radiology. However, in order for such systems to perform adequately, large amounts of training data are required. These data should ideally be standardised and of adequate quality to allow for further usage in training of artificial intelligence algorithms. Unfortunately, in many current clinical and radiological information technology ecosystems, access to relevant pieces of information is difficult. This is mostly because a significant portion of information is handled as a collection of narrative texts and interoperability is still lacking. This review aims at giving a brief overview on how structured reporting can help to facilitate research in artificial intelligence and the context of big data.

## Key points

- Research in artificial intelligence and big data heavily depends on the available data
- Today, most radiological report data are only available as unstructured narrative text
- Structured radiological reporting has the potential to facilitate research and development in machine learning for radiological applications
- Interoperable standards for structured reporting are available
- Implementation of structured reporting in clinical routine is still scarce

## Background

The development of a machine learning (ML) or, in the broader context, an artificial intelligence (AI) algorithm is often compared to the learning experience of a child [1, 2]. During early childhood, parents are likely to repeat many times that a specific object is a ball or a dog until, finally, the child confidently identifies these objects in her/his environment. Intuitively, we refrain from putting too much effort into immediately differentiating various breeds of dog or types of ball until the child gets older. Also, we structure our communication by using a simple but consistent vocabulary and referencing the objects unambiguously (i.e. by pointing at a dog or holding a ball while naming the object).

Imagine that, instead of correctly referencing objects and using simple, standardised terms, parents would only spell out a dog’s breed name (and never mention the word ‘dog’ itself) and sometimes randomly point at the wall instead of the dog. It is evident that a child presented with this kind of information would take substantially longer time to make sense of this information until he could correctly identify a dog as a dog.

This concept of early childhood learning, with all its potential challenges, can be easily translated to the current ‘hot’ topics in radiology, i.e. big data, ML, and AI, in order to help better understand why and how data needs to be structured to be able to support further developments and application of advanced computer systems in radiology.

Similar to a developing brain, the development of AI algorithms needs massive amounts of training data, ideally highly specific data, correctly referenced and provided in as structured a form as possible. Of course, the data requirements heavily depend on the task to be solved. While there are some simple tasks, such as using linear regression to predict a target variable out of three input variables, which can be solved with a limited amount of training data, other tasks, such as finding and correctly classifying brain haemorrhage in a computed tomography (CT) scan of the head, can be much more challenging.

In this review article, we provide an overview of recent developments in big data and ML in radiology with a particular focus on why and how structured reporting could play a crucial role for the future of radiology.

## Unstructured data in radiology

In theory, massive amounts of training data for the development of complex AI algorithms should be available in healthcare and radiology. For example, assuming that an average-sized radiological department performs 200 CT scans per year for the detection of pulmonary embolism of which around from 25 to 50 show a visible thrombus in the pulmonary arteries, over the course of 10 years this would amount to a total of at least 2000 scans with at least 250 showing pulmonary embolism. These imaging exams could be made accessible through the department’s Picture Archiving and Communication System (PACS) and should be accompanied by the corresponding radiological reports with at least one clear statement regarding the presence or absence of a pulmonary embolism.

One would expect that the most difficult part could be to develop an algorithm able to detect the emboli within the pulmonary arteries automatically. However, considering recent technological advances, it appears that this could be solved relatively easily if sufficient accurately labelled data were available. However, access to accurately labelled data is problematic. The vast majority of radiological reports are currently written as unstructured narrative text and extraction of information contained in such unstructured reports is challenging.

Natural language processing (NLP) has made substantial improvements in the last decades and could in theory help to mine unstructured reports for relevant information [3–5]. However, one crucial challenge remains for NLP algorithms. In a large number of cases, conventional radiological reports show a considerable amount of variation not only in language but also in the findings reported. In some clinical settings, examinations are highly specific and reported findings relatively consistent allowing for accurate classification of the report content. One such case is CT for pulmonary embolism where the excellent accuracy of classification could be shown [6]. However, in other examinations, such as magnetic resonance imaging (MRI) of the lumbar spine, there is such a marked variability in interpretative findings reported that accurate classification of report content is highly unlikely [7]. This problem is even more aggravated when the radiologist’s impression of a particular examination also considers other clinical information that is not included in the radiological report.

**Fig. 1:**
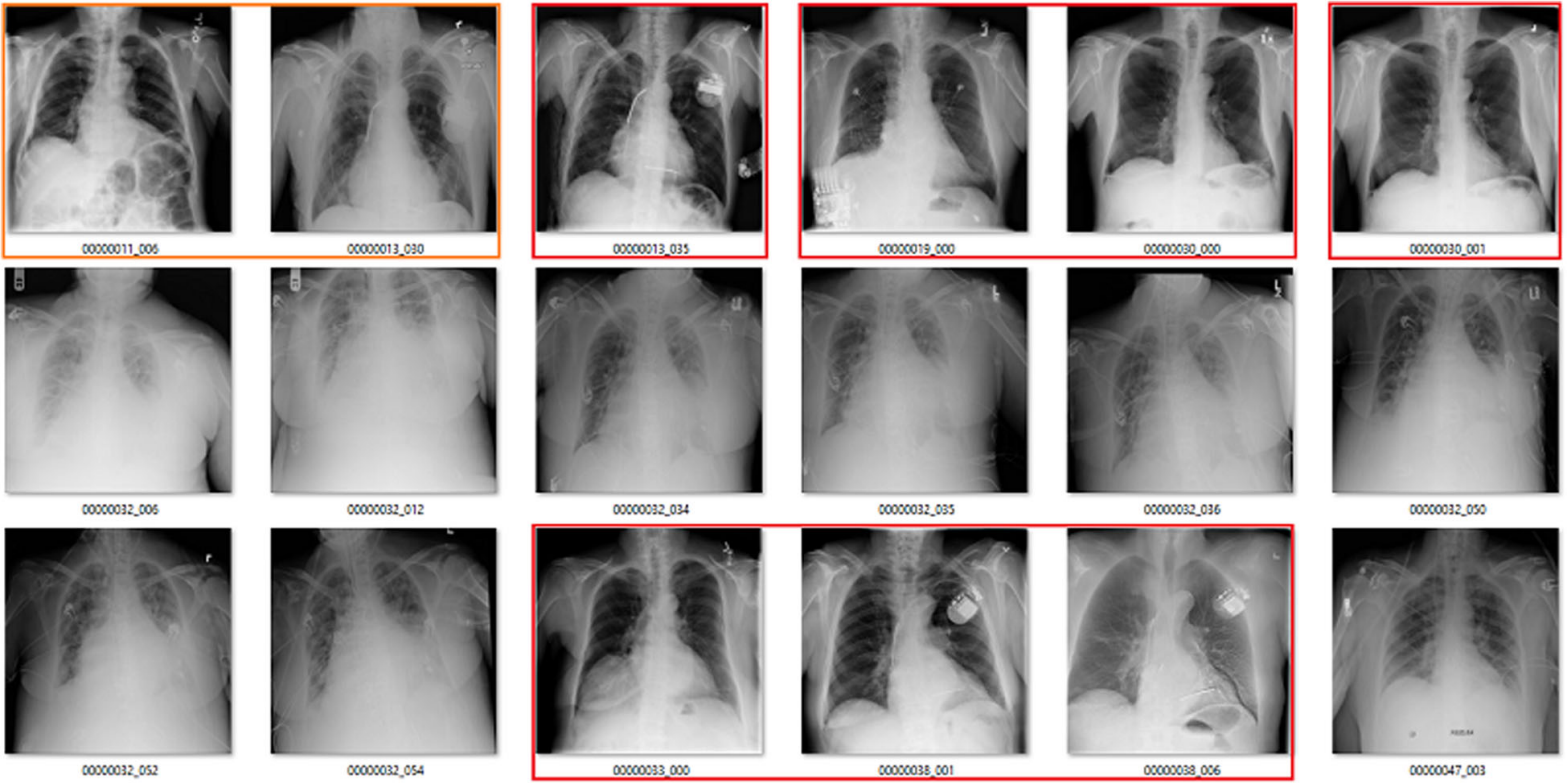
Images from the ChestXray14 dataset labelled as showing atelectasis (red boxes indicate wrong label, orange indicate doubtful). Courtesy of Luke Oakden-Rayner (available at: https://lukeoakdenrayner.wordpress.com/2017/12/18/the-chestxray14-dataset-problems/) , with permission

This was most notably demonstrated by a study published by a group from Stanford University under the lead of Andrew Ng, that, in its first version [8], claimed that an algorithm showed superhuman performance in recognising pneumonia on plain chest radiography, This claim was quickly picked up and echoed through various media sources [9]. However, there was a crucial issue with the original training dataset for which labels had been extracted using text-mining techniques [10]. Among other issues, there was a certain proportion of images with wrong labels as well as overlap between different labels such as consolidation, atelectasis, infiltration, and pneumonia (Fig. 1). Moreover, while all of these findings may have a similar visual appearance, to diagnose pneumonia, usually clinical information and laboratory results are taken into consideration which may not be included in the final report. The initial claim that the algorithms showed superhuman performance in the detection of pneumonia has since been put into perspective taking into account these issues [11].

Nevertheless, this prominent example clearly shows that when developing AI algorithms, the most critical step is to extract meaningful and reliable labels to establish a valid ground truth. At this point, it seems evident that similar problems will potentially always be encountered when using unstructured radiological reports as the basis for extracting labels. In this context, structured radiological reporting could offer a solution through standardised report content and more consistent language. Also, apart from being difficult to analyse for automated systems seeking to extract knowledge from the reports, it has been shown that referrers also favour more structured radiological reports [12–16]. Moreover, various studies showed that structured reports showed greater clarity and greater completeness with regard to relevant information than unstructured reports [17, 18].

## Structured reporting in radiology

Following the growing evidence that structured reports would have a beneficial impact on the communication between radiologists and referring physicians, the Radiological Society of North America (RSNA) presented its *Reporting Initiative* aimed at developing and providing vendor-neutral reporting templates [19, 20]. This was later picked up by *Integrating the Healthcare Enterprise* (IHE) and led to the publication of the *Management of Radiology Report Templates* (MRRT) profile [21]. This profile extensively describes the concepts and technical details for interoperable, standardised, structured, report templates. Report templates are developed and provided using a subset of standard Hypertext Markup Language (HTML) and allow for the integration of RadLex terms. This offers the possibility of further standardising report template content as synonyms and even content in different languages could be mapped to the same RadLex code [22].

Major radiological societies already offer collections of such MRRT-compliant report templates. For example, the RSNA Reporting Initiative offers more than 250 report templates on its radreport.org website. There has also been a memorandum of understanding signed between the European Society of Radiology and the RSNA, leading a joint *Template Library Advisory Board* and an open platform where European users can upload and discuss their report templates (open.radreport.org) [23]. Other societies have also published various consensus statements providing disease-specific report templates which have been shown to provide the most benefit to referring physicians in most cases [24]. For example, the American Society of Abdominal Radiology published a consensus statement regarding reports for pancreatic ductal adenocarcinoma, and the Korean Society of Abdominal Radiology published a similar paper with regards to rectal cancer MRI [25, 26].

Nevertheless, integration of structured radiology reporting is still scarce in clinical routine. Some vendors (*e.g.,* Nuance, Burlington, MA, USA) provide the possibility of integrating such report templates into their speech-recognition solution. However, even in these cases reports are composed using report templates but then stored as plain text in the hospital or radiology information system. Although this surely allows for more easy text mining due to more standardised reports, storing the respective parts of information from the report templates in a dedicated database as discrete bits of information would allow for even easier access to these data.

**Fig. 2:**
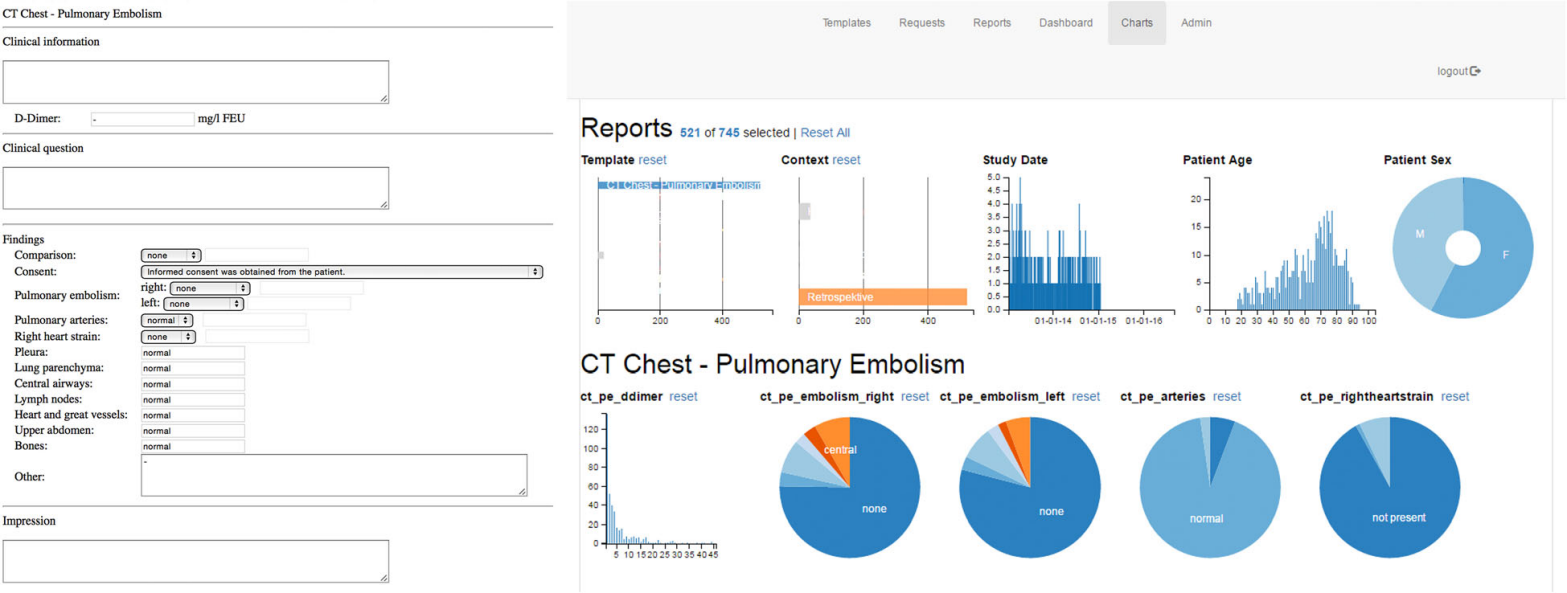
Example of a structured report template for pulmonary embolism (left), and a dashboard visualising summary results of all reports created with this template (right). Such information could also be used as labels to the corresponding imaging study

Initial prototype applications have been described that support IHE MRRT-compliant report templates and take full advantage of the concept of structured reporting by storing report content in a dedicated database [27]. Such implementation allows for easy extraction and further processing of data contained in the radiological reports (Fig. 2). This information can then be used for various purposes, ranging from simple epidemiological statistics to using the data as easily accessible and accurate ground truth for the training of AI algorithms [28]. Other vendors are actively developing similar tools (*e.g.,* Aycan, Würzburg, Germany) for providing cloud-based solutions that use proprietary template formats (*e.g.,* Smart Reporting, Munich, Germany).

## Digitised workflows, big data, and artificial intelligence

The shift from an analogue film-based workflow to fully digitised radiology departments has undoubtedly been a major change to radiological practice. It can be expected that the next significant impact on radiology and healthcare, in general, will come from the integration of big data and AI applications [29–31].

Currently, one of the main challenges for the development of AI solutions for healthcare and radiology remains the unstructured nature of the data stored in electronic health records and hospital information systems. Of course, to a certain extent, big-data techniques allow for working with unstructured data, but depending on the task at hand *e.g,* pattern recognition/detection of pathologies in radiological images, uncertainties introduced by unstructured data, as mentioned above, can have a significant impact on the algorithm’s performance. Nevertheless, there are already tasks where these uncertainties can be tolerated or where metadata (which are often stored in more structured ways) can be used to improve a department’s workflows and thus positively impact patient care. For example, studies have shown that using ML and big-data techniques on metadata extracted from an institution’s PACS and hospital informatio system enables accurate prediction of waiting times and potential no-shows in a radiological department [32, 33]. Such insight into department workflows could prove to be of high value and while some uncertainties remain as to which factors influence waiting times, recognising patterns in the data may allow identifying potential causes which then could be further investigated. Other studies showed that an automated lung nodule registry tracking system was able to significantly reduce the tracking failure rate for incidental pulmonary nodules [34].

## Conclusion

The examples mentioned above demonstrate that there are many potential applications of big-data techniques and AI in radiology. Interest in these topics has increased sharply over the past few years, and there has been a marked increase in publications about AI algorithms in radiology. Most publications focus on image interpretation, while only a few target on other parts of the diagnostic radiology workflow.

In the development of AI algorithms for radiology and even healthcare, in general, it is clear that the data used to train and validate the algorithms is one of the most important aspects to be taken into account. Consequently, requirements for these data largely depend on the question that is to be answered. More straightforward tasks may be sufficiently addressed using unstructured data or metadata, while other tasks, such pattern or disease recognition using computer vision techniques, may require more accurate ground truth than can currently be extracted from unstructured radiology reports.

Comparable to early childhood learning, AI algorithms ideally need large amounts of data with accurate and consistent labels to attain high performance in visual recognition tasks. Due to their high degree of variability in content and language, conventional narrative radiological reports pose a vital challenge to extract meaningful and reliable information that could be used for AI algorithm development.

Structured reporting has been deemed the fusion reactor for radiology [35]. Especially in the context of AI, it is evident that information extracted from such template-based structured reports could offer a relevant benefit. Currently, the establishment of a reliable ground truth is cumbersome. Either information is extracted from narrative reports using NLP techniques, introducing some degree of uncertainty, or time-consuming manual review by human readers is required. Understandably, both approaches have their limitations. It would, therefore, be desirable to integrate structured reporting into clinical routine, allowing for more accessible and standardised information from radiological reports, ideally while also improving report quality and without causing additional workload for the individual radiologist. Radiologists should, therefore, ask vendors to provide practical solutions to implement structured reporting in their routine workflow, as this could prove to be the key for further developments of AI in radiology.

## Abbreviations

AI: Artificial intelligence
CT: Computed tomography
IHE: Integrating the Healthcare Enterprise
ML: Machine learning
MRI: Magnetic resonance imaging
MRRT: Management of Radiology Report Templates
NLP: Natural language processing
PACS: Picture Archiving and Communication System
RSNA: Radiological Society of North America
